# Factors Associated with Parenting Adaptability in Facing the First COVID-19 Lockdown: A Study on Portuguese Parents

**DOI:** 10.3390/healthcare11202745

**Published:** 2023-10-16

**Authors:** Ana P. Antunes, Silvana Martins, Ana T. Almeida

**Affiliations:** 1 Department of Psychology, Faculty of Arts and Humanities, University of Madeira, Campus Universitário da Penteada, 9020-105 Funchal, Portugal; aantunes@uma.pt; 2 Research Centre on Child Studies, Institute of Education, University of Minho, Campus de Gualtar, 4710-057 Braga, Portugal; 3 ProChild CoLAB Against Poverty and Social Exclusion Association, Campus de Couros, Rua de Vila Flor, 4810-225 Guimarães, Portugal; silvana.martins12@gmail.com; 4 Health Sciences Research Unit, Nursing School of Coimbra, Avenida Bissaya Barreto, Polo C, 3046-851 Coimbra, Portugal

**Keywords:** positive parenting, family resilience, family dynamics, COVID-19 lockdown, daily routines, co-parenting, emotional experience, support network, well-being

## Abstract

Parenting was drastically challenged during the COVID-19 pandemic as families complied with the consequent containment for sanitary and social reasons. The purpose of this study is to explore the relationships among self-report measures (co-parenting, emotional experience and parental support network) and the employment condition (teleworking parents and at-home parents with governmental leave aid) that might be associated with the everyday life change, and their contribution towards family’s daily routines during the first COVID-19 lockdown. A sample of 878 parents (90.2% mothers), aged from 21 to 61 years (39.58 ± 6.08), and mainly of Portuguese nationality (97.2%), was gathered through an online survey. Participants completed sociodemographic data and answered questions related to four positive parenting dimensions (Daily routines, Co-parenting, Emotional experience, and Support network) rating on a five-point Likert scale how much their behavior changed during the lockdown. Descriptive statistics and multilinear regression models were tested as well as a mediation model. The results showed that Emotional experience, Co-parenting, and Support network played an important role in family Daily routines, reinforcing that the first COVID-19 lockdown was lived differently by parents according to their employment conditions. Furthermore, the pathways model of factors associated with parental adaptability is suggestive that the direct effect of Emotional experience on Daily routines is partially mediated by Co-parenting and Support network. In line with previous studies on the complexity and dynamic ecology of parenting, the reported indicators showcase the interrelated facets of parenting and its corresponding support needs. Further, clear indicators for the design of family support interventions and family resilience enhancement to stressor events are discussed.

## 1. Introduction

Parenting is a challenging task, especially in times of crisis when families faced the COVID-19 public health threat worldwide. However, above and beyond the turmoil, the lived changes that the pandemic evoked also capitalized families’ resources and, ultimately, coping with the strain and stress promoted their resilience. In the perspective of Froma Walsh [1], facing stressful moments, which the COVID-19 pandemic was, demanded the activation of resilient family processes that, despite the crisis, could be opportunities for the development of creative solutions, new dynamics for problems’ resolutions and states of perseverance to go through hard experiences. In this regard, burgeoning evidence on the systemic processes of parenting and its outcomes associate the fulfillment of parents’ developmental potential to everyday experience that can influence both parents’ and children’s well-being [2].

According to Rodrigo [3], parenting is currently viewed as an early evolving and complex task conveying three interrelated facets: (a) dyadic parenting, the basis for attachment and parental responsivity to the child’s needs from early caregiver–infant interactions throughout infant years and after to boost healthy, stable and protective bonding; (b) team parenting, the fabric for carrying out a structured environment offering support, stimulation, learning, rewarding and daily guidance; and (c) social parenting, the net of surrounding supporters built on the parent’s informal and formal relations to meet his or her corresponding support needs. In this regard, the parenting task is not solely confined to the parent–child dyad, but it comprises other significant co-parental alliances (e.g., grandparents or ex-partners), providing complementary support to facilitate the family–work balance and the housework parity; and, rather essentially, it also entails a bulk of resourceful components from personal social relationships (e.g., relatives, friends or neighbors) to those accessible at different community services (education, social welfare, health, security, legal institutions) and even, more recently, online support (e.g., public websites, NGO’s institutions, and social media). Moreover, the growing social awareness concerning the parents’ contribution to the children’s well-being and their optimal development has known an extraordinary change in the family and parenting support domain. Particularly, the growth and expansion in the provision of care across the diverse array of community services has also nurtured the sense of a shared social responsibility to the parents’ support needs [2]. At the parents’ side, the counterpart is an increased entrust to partner with the community aiming at promoting the quality of family’s context, the family and child participation and sustained development to their social inclusion and cohesion [3].

Worldwide, published studies about parenting during the COVID-19 pandemic illustrate that parents and children’s personal and dyadic processes as well as all-enveloping external factors contribute to adaptative changes on parenting and family dynamics. In fact, the pandemic experience is often related as a moment of crisis (due to restrictions and changes in daily life, emotional distress, work and finances, pandemic management and communication, societal impact, COVID-19 and other health issues) but also as a moment of opportunities (reflection and growth, reach for meaningful/enjoyable activities, benefits at an interpersonal level, and other noteworthy themes—digitalization and environmental effects) [4]. Yet, studies do not underestimate that risk and resilience have both presented challenges for parental emotional regulation. In a study with participants from 25 countries, the authors found that emotional regulation moderated the association of parental stress (general and parenting stress) with parental burnout. Particularly, rumination increased that association, whereas cognitive reappraisal decreased it [5]. In Israel, a study revealed that children presented more stress reactions when parents exhibited more difficulties in emotional regulation and where exposed to higher stress. It is important to mention that only fathers’ playfulness was related inversely to children’s stress [6]. In the same line, a study from Canada reported that parental emotional regulation was an important parental resource to the decrease in stressors perception [7]. A comparative study of Portuguese and British families revealed that unrealistic parental demands (schoolwork and daily routines) and emotional dysregulation were manifested differently [8]. There, the Portuguese parents showed higher results than British parents concerning those dimensions, but that differences on parenting were not associated with significant differences on children’s adjustment (quality of life and anxiety).

Along with parental self-regulation variables, the impact of the lockdown on Portuguese children was also associated with parental competency perceptions and parent–child interactions [9]. Yet, intrapersonal capacities that enable parents’ adaptative responses to parenting in novel and challenging conditions ought not be singled out from the multiple sources and forms of family support. Among other relevant data on this issue, a Chinese study revealed that family support and co-parenting could reduce the daily parenting hassles and, in consequence, protect the mental health of parents [10]. Still, another study, in Turkey, reported that parental self-efficacy and marital adjustment decreased when the perception of stress increased [11]. They also found that the intrafamilial support contributed to higher family resilience than external family support. Confirming the critical significance of couple relations to parent’s support and well-being during the lockdown, another study conducted in China showed that family functioning was strongly affected by couple relationships followed by other stressors (family income and mental health) while parent–child conflict weighed comparatively less in family functioning and the same diminishing effect was perceived regarding community network and resources [12]. Indeed, the pandemic period and its consequent lockdown was also regarded as a period of positive family moments, eliciting memorable experiences, thereby fostering family resilience. Likewise, parents referred to that time as an opportunity to experiment with new family routines and to closely watch the development of their children [9,13,14]. Families also stated that the lockdown allowed the development of family bonding, the flourishing of new family resources and stable and secure routines, which constituted a protective factor against the adverse lived experience [15]. Interestingly, among Portuguese parents, some revealed heightened concerns around the difficulty of work–life balance and the need for stress management while others valued the opportunities to foster parent–child interactions, more time and proximity which, in turn, had a protective influence on the family’s well-being [13,14,16]. This is borne out consistently when studying family adaption in relation to parents’ different work conditions. Unprecedented challenges were met by Portuguese teleworking parents who mentioned more difficulties in parent–child activities, co-parenting, and parental agreement compared to at-home parents caring for children with governmental aids, which generally revealed more positive parenting behavior changes concerning daily routines, positive discipline, parent–child activities, and enriched environment [13]. At-home teleworking parents also revealed more changes concerning stress management than other parents to face lockdown demands [13]. Actually, parent’s work conditions had different impact on parenting. Yet, how parents parented was tied to different family dynamics whereby a constellation of factors did not affect parents equally. For instance, where emotional responses and parent’s psychological adjustment (e.g., distress, humor, anxiety mood) had a stronger impact on parental practices such as punishment versus positive discipline, nurturing and parent–child closeness appeared to b more stable and not so permeable to transitional hazards, thereby promoting positive parent–child communication and playful activities [17,18].

Applying the systemic approach to address changes in parenting during COVID-19 pandemic, the purpose of this study was to deepen our understanding regarding the associations among the parent’s emotional experience, parenting daily routines, co-parenting and support network. Additionally, in line with the already published study, a secondary goal was identifying the different employment conditions associated with everyday life changes in parenting dimensions (Co-parenting, Emotional Experience and Support Network) and their contribution towards family’ daily routines during the first COVID-19 lockdown. Specifically, the hypotheses tested in this study consider the following assumptions:

**Hypothesis** **1.** *Positive changes in the parent’s emotional experience (i.e., higher levels of emotional sensitivity, better emotional regulation and stress management responses and less emotional tension) would be associated with more positive changes in daily routine (more instances of positive discipline, nurturing, enriched environment and parent–child activities)*.

**Hypothesis** **2.** *Positive changes in co-parenting and support networking would enhance the parent’s emotional experience, yielding a mediational effect on parenting adaptability in the daily routine*.

## 2. Materials and Methods

### 2.1. Study Design

The present study is part of a Portuguese study (Parenting in COVID-19 times) of which some results have already been published [13]. The design of this study was a cross-sectional survey to analyze the association between variables, namely the predictive variables and the mediator variables.

### 2.2. Sampling

The participants were selected from a larger sample of parents (1384 parents), and they were selected because they were the group of parents who manifested more changes in family dynamics during the period of the first COVID-19 lockdown according to a previous study [13]. In fact, they were selected from four groups of parents (at-home parents with children under 12 with a licensed work leave and governmental aids, at-home parents who were teleworking, parents working out-of-home as usual, and parents who were unemployed), because they generally manifested more changes in the analyzed Parenting Dimensions (more details about that can be seen at [13]).

So, for the main purpose of this study (to analyze whether Employment condition and Co-parenting, Emotional experience, and Support network predict changes in parenting concerning Daily routines) we selected these parents because they revealed more changes in their family dynamics [13], and we wanted to study in depth the contribution of the studied variables to that change. Consequently, in addition to being confined at home, they met one of two other possible conditions: (a) benefited from a specific licensed work leave under COVID-19 governmental aids to parents with children under 12; or (b) stayed home teleworking.

Also, to be included in the study, participants were either a parent or a substitute caregiver of one or more children 18 or under, living at home with them.

### 2.3. Measures

An ad hoc questionnaire was used to collect the data (the same described at Antunes et al. [13]). The questionnaire was intended to ask parents about their parenting and family dynamics. The questionnaire collected some sociodemographic information (e.g., gender, age, job statuses, number of children, age of children, and number of days in lockdown) and was formed by five scales (Daily routines, Co-parenting, Emotional experience, Support network and Parental mood) and an open question (Would you like to mention any situation that has impacted you and your family during the lockdown?). In the present study, the Parental mood scale and the open question were not used.

The four scales (Daily routines, Co-parenting, Emotional experience, Support network) are briefly described and the Cronbach’s alpha and the McDonald’s omega, which were calculated for this sample, are also presented (additional information about the scales, the confirmatory factor analysis, and the internal consistency coefficient can be seen in Antunes et al. [13]):

(a) The Daily routine scale (15 items) assesses the degree of change on basic care in daily chores (hygiene, meals, sleep hours, shared and nonshared activities, limits, and guidance). This scale has four subscales: positive discipline (e.g., item 3, “I maintain my children’s wake times and bedtimes during school days”); parent–child activities (e.g., item 8, “I am available to supervise my children’s school work”); nurturing (e.g., item 2, “I make my children take responsibility and help out with household chores”); and enriched environment (e.g., item 15, “I plan at least one different activity for the weekend”). For this sample, the Cronbach’s alpha was 0.815 and McDonald’s omega was 0.815.
(b) The Co-parenting scale (11 items) assesses marital involvement and mutual support in multiple childcare duties and has two subscales: parental alliance (e.g., item 1, “We decide how to solve our children’s discipline problems together”) and parental agreement (e.g., item 11, “We point out each other’s good qualities to our children”). For this sample, the Cronbach’s alpha was 0.884 and McDonald’s omega was 0.886.
(c) The Emotional experience scale (14 items) assesses emotional experience during the lockdown period and has four subscales: emotional sensitivity (e.g., item 2, “I pay attention to what my children say and how they feel”); emotional tension (e.g., item 7, “I recognize that it does not take much for me to run out of patience”); emotional regulation (e.g., item 6, “I have had to be extra patient when taking care of several things at the same time”); and stress management (e.g., item 9, “I try to control myself so I do not pass on my worries to my children”). For this sample, the Cronbach’s alpha was 0.816 and McDonald’s omega was 0.788.
(d) The Support network scale (nine items) assesses the changes in the support of formal and informal networks provided to the family during lockdown and has two subscales: informal network (e.g., item 1, “I feel like my family and friends care about my children”) and formal network (item 5, “I sense that educators/teachers care whether my children learn”). For this sample, the Cronbach’s alpha was 0.843 and McDonald’s omega was 0.829.

The responses of the four scales were rated on a 5-point scale ranging from 1 to 5, reflecting the degree of change (1 for “happens much less often”, 2 for “happens less often, 3 for “happens the same”, 4 for “happens more often”, and 5 for “happens much more often”). A score of zero was included to allow the participants to check whenever the item(s) did not apply and these values were coded as missing.

### 2.4. Data Collection

The present study is part of a Portuguese study (Parenting in COVID-19 times) that was approved by the Ethics Commission for Research in Social and Human Sciences of the University of Minho (reference CEICSH045/2020 of 13 May 2020).

To perform the study, an ad hoc questionnaire was constructed and made available through an online platform. At the beginning of the questionnaire, the participants acknowledged the purpose and procedures of the study, the assurance of confidentiality and voluntary participation, and their consent to participate in the study was mandatory to continue the filling of the questionnaire.

Parents were recruited trough social media platforms like Facebook and WhatsApp.

The study was conducted by asking the participants about to the first lockdown in Portugal (the state of emergency was decreed from 18 March to 2 May and the state of calamity from 3 May to end of July, where some general lockdown measures were cautiously removed). The data were collected from 15 May to 26 June, at the end of the academic year while several restrictive measures, including lockdowns, remained in force.

### 2.5. Data Analysis

Statistical analyses were conducted using SPSS, version 28.0 (IBM Corp. Released 2020. IBM SPSS Statistics for Windows, Version 28.0. Armonk, NY, USA: IBMCorp) and JASP 0.16.0.0 for Windows. The internal consistency reliability study was done through analysis of the Cronbach’s alpha and McDonald omega [19,20]. Descriptive statistics were calculated as means, standard deviations, and ranges. The association with dependent variables, as well as mediator and predictor variables were also analyzed. Pearson correlation coefficients were calculated to study the relation between different variables (the categorical sociodemographic variable Employment condition, which included teleworking parents and at-home parents with government licensed work leave; and the results of scales and subscales). Multiple linear regressions were performed to study the relations between an outcome variable (the four subscales of Daily routine: Positive discipline, Parent–child activities, Nurturing, and Enriched environment) and other predictor variables (Employment condition, and the subscales of Co-parenting, Emotional Experience and Support network). The predictor Employment condition was transformed into a dummy variable where zero corresponded to teleworking parents and one corresponded to at-home parents with government licensed work leave. Principal assumptions to linearity justify the use of linear regression models abiding normality of the errors, homoscedasticity, and normality of the error distribution. For this study, the assumptions of linear regression analysis, values of inter-correlations, the Durbin–Watson and Variance Inflation Factor (VIF) were not violated. In multivariable regression analyses, the standardized beta coefficient (Beta) compares the strength of the association between the predictors and the outcome variables introduced in the model. All of the statistical assumptions were checked [21]. Contribution of the independent variable in the model is expressed as explained variance (adjusted R^2^).

The mediation model was calculated using the statistics program JASP version 0.16.0.0. Emotional experience was included as a predictor, social support and co-parenting as the mediators, and daily routine as the outcome. Parent’s employment was not included because it was a categorical variable. Direct, indirect, total indirect and total effects were calculated with bootstrap confidence interval based on 1000 resamples.

The level of significance was set to *p* < 0.05.

## 3. Results

### 3.1. Demographics

The participants in the study were 878 parents (90.2% mothers), with ages from 21 to 61 years old (M = 39.58, SD = 6.08) and were mainly of Portuguese nationality (97.2%).

Most of the participants was married or had a stable relationship (78.2%) and had a university degree (79.7%). The majority had one child (41.2%) or two children (46.8%) living at home (the number of kids ranged from one to five). The age of the children was between one year old to 18 years old (M = 7.00, SD = 4.65) and most of the children revealed no special educational needs (88%).

The participants experienced on average 66.4 days of lockdown, ranging from zero to 122 days in lockdown (SD = 15.1).

All parents of this sample were employed, but a group of them were at home teleworking (67.7%) and the other group was at home, on a licensed work leave and receiving governmental assistance (32.3%), to care for their children.

### 3.2. Descriptive Statistics

Table 1 presents the means, the standard deviations and ranges for the Daily routine, Co-parenting, Emotional experience, and Support network and their subscales. Generally, the range of results was between 1 (“happens much less often”) and 5 (“happens much more often”), except for Daily routine, Positive discipline, Nurturing and Emotional experience, whose lower results were from 1.77; 1.5; 2; and 1.75, respectively. The average results in the scales and subscales were between 3.02 and 4.12, suggesting average results between 3 (“happens the same”) and 4 (“happens more often”). The subscales Parent–child activities, Nurturing, Emotional sensitivity, Emotional tension, and Stress management presented the highest mean values. The results suggest that the responses of the participants tended to be between “happens the same” (3) and “happens more often” (4), indicating that the parents experienced changes in these domains during the lockdown.

### 3.3. The Pearson Correlation Coefficients between Employment Condition, Scales and Subscales

Table 2 displays the Pearson correlation coefficients between the employment condition, daily routine, co-parenting, emotional experience and support network scales and subscales.

The results showed moderate positive correlations for daily routine and co-parenting (r = 0.521, *p* < 0.01), parental agreement (r = 0.516, *p* < 0.01), parental alliance (r = 0.415, *p* < 0.01), emotional experience (r = 0.396, *p* < 0.01), emotional sensitivity (r = 0.546, *p* < 0.01), emotional regulation (r = 0.515, *p* < 0.01), support network (r = 0.30, *p* < 0.01) and informal network (r = 0.328, *p* < 0.01).

Moderate positive correlations were found for positive discipline and co-parenting (r = 0.488, *p* < 0.01), parental agreement (r = 0.475, *p* < 0.01), parental alliance (r = 0.412, *p* < 0.01), emotional experience (r = 0.317, *p* < 0.01), emotional sensitivity (r = 0.458, *p* < 0.01), emotional regulation (r = 0.409, *p* < 0.01) and informal network (r = 0.306, *p* < 0.01).

Moderate positive correlations were found for parent–child activities and co-parenting (r = 0.342, *p* < 0.01), parental agreement (r = 0.334, *p* < 0.01), emotional sensitivity (r = 0.308, *p* < 0.01) and emotional regulation (r = 0.371, *p* < 0.01).

Moderate positive correlations were found for nurturing and emotional experience (r = 0.373, *p* < 0.01), emotional sensitivity (r = 0.403, *p* < 0.01), and emotional regulation (r = 0.311, *p* < 0.01).

Moderate positive correlations were found for enriched environment and co-parenting (r = 0.344, *p* < 0.01), parental agreement (r = 0.354, *p* < 0.01), emotional experience (r = 0.301, *p* < 0.01), emotional sensitivity (r = 0.4116, *p* < 0.01), and emotional regulation (r = 0.374, *p* < 0.01).

No statistically significant correlations were found for the employment condition and daily routine, co-parenting, emotional experience and support network scales and subscales.

### 3.4. Multivariate Linear Regression Analysis

In this section are presented the results from the multivariate linear regression analysis exploring the co-parenting, emotional experience, and support network subscales as predictors of the daily routine subscales (positive discipline, parent–child activities, nurturing, and enriched environment).

#### 3.4.1. Positive Discipline

For positive discipline (Table 3), the model obtained was statistically significant (F (9, 670) = 35.579, *p* < 0.001). The results indicated that the model could explain 31.4% of the positive discipline changes (R^2^ = 0.323, adjusted R^2^ = 0.314). Parental agreement (β = 0.281, *p* < 0.001), emotional sensitivity (β = 0.188, *p* < 0.001), emotional regulation (β = 0.119, *p* = 0.004) and informal network (β = 0.116, *p* = 0.001) influenced the changes in positive discipline. More changes in parental agreement, emotional sensitivity, emotional regulation and informal network produced more changes in positive discipline.

#### 3.4.2. Parent–Child Activities

For parent–child activities (Table 4), the model obtained was statistically significant (F (9, 670) = 22.268, *p* < 0.001). The results indicated that the model could explain 22% of the parent–child activity changes (R^2^ = 0.230, adjusted R^2^ = 0.220). Employment condition situation (β = 0.107, *p* < 0.002), parental agreement (β = 0.169, *p* < 0.001), emotional tension (β = −0.169 *p* < 0.001) and emotional regulation (β = 0.311, *p* < 0.001) influenced the changes in parent–child activities. At-home parents with government licensed work leave, more changes in parental agreement and emotional regulation and less changes in emotional tension produced more changes in parent–child activities.

#### 3.4.3. Nurturing

For nurturing (Table 5), the model obtained was statistically significant (F (9, 669) = 17.099, *p* < 0.001). The results indicated that the model could explain 17.6% of the nurturing changes (R^2^ = 0.187, adjusted R^2^ = 0.176). Parental agreement (β = 0.140, *p* = 0.004), emotional sensitivity (β = 0.233, *p* < 0.001), emotional regulation (β = 0.109, *p* = 0.017) and stress management (β = 0.124, *p* = 0.003) influenced the changes in nurturing. More changes in parental agreement, emotional sensitivity and stress management produced more changes in nurturing.

#### 3.4.4. Enriched Environment

For enriched environment (Table 6), the model obtained was statistically significant (F (9, 668) = 22.491, *p* < 0.001). The results indicated that the model could explain 22.2% of the enriched environment (R^2^ = 0.233, adjusted R^2^ = 0.222). Employment condition (β = 0.101, *p* < 0.003), parental agreement (β = 0.218, *p* < 0.001), emotional sensitivity (β = 0.170, *p* < 0.001), emotional regulation (β = 0.152, *p* < 0.001) and informal network (β = 0.141, *p* < 0.001) influenced the changes in enriched environment. At-home parents with government licensed work leave, and more changes in parental agreement, emotional sensitivity, emotional regulation, and informal network produced more changes in enriched environment.

### 3.5. The Mediation Model

The hypothesized mediation model that was constructed, and consequently tested, revealed a direct effect of the Emotional experience in Daily routines, and Support networks and Co-parenting as partial mediators between Emotional experience and Daily routines.

**Figure 1:**
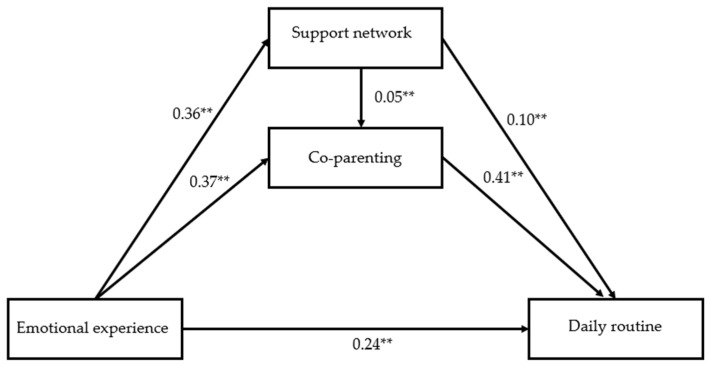
The serial multiple mediation of support network and co-parenting between emotional experience and daily routine. Note: Path coefficients were shown in standardized regression coefficients. ** *p* < 0.001.

The results of the mediation of co-parenting and support networking between emotional experience and daily routine are described in Table 7 and Figure 1. The proportion of variance explained for each of the predictor variables in the mediation model is 7% (R^2^ = 0.07) for support network and 11.4% (R^2^ = 0.114) for co-parenting. The total effect is 0.43 (*p* < 0.001), the direct effect is 0.24 (*p* < 0.001) and the total indirect effect is 0.19 (*p* < 0.001). In addition, both indirect effects are statistically significant: emotional experience → support network → daily routine and emotional experience → co-parenting → daily routine. Support network and co-parenting are partial mediators between emotional experience and daily routine.

## 4. Discussion

The main purpose of this study was to analyze the relations among the different factors associated with adaptative parenting (Co-parenting, Emotional Experience and Support Network). The second aim resulted from an extension of previous findings with this group of parents seeking to identify whether the impact of the parents’ employment condition was also perceived in daily routine dimensions, namely predicting the changes in parenting adaptability during the first COVID-19 lockdown. As can be seen, the results allowed us to state that the aims of the study were achieved and its main contributions are discussed next.

Regarding the first purpose, the results revealed a rather homogenous and stable pattern of association among daily routine changes with co-parenting changes and emotional experience. These changes are extensive to positive discipline, parent–child activities, nurturing and enriched environment. In common, the four sub-dimensions of daily routine indicate associations with observed changes in the parental agreement dimension and emotional regulation. Indeed, no matter the singularity of supplementary associations with other sub-dimensions of co-parenting (parental alliance) and emotional experience (e.g., emotional sensitivity, emotional tension), the predominance of this pattern presents an effortful conscience of balance and control needs, which is pivotal to parental functioning in competent ways. These findings align with other studies where couple relationships changed during the pandemic and were considered a key factor to discuss parenting and family dynamics, excepting single-parent families [10,11,12,22]. Rather important is the association of emotional regulation reinforcing the control of parent’s stress [5] and, consequently, child stress [6]. Worth mentioning is the association of nurturing with emotional experience, emotional sensitivity, and emotional regulation. These results seem to be in line with the idea that changes in nurturing were associated with changes in emotional factors related to parents’ competence to regulate their emotions and respond to their children’s needs [7].

Additionally, daily routine was associated with the total support network and, more specifically, to an informal network, which was associated with positive discipline and an enriched environment. Conversely, formal network was not associated with the daily routine scale nor with its subscales, possibly reflecting the social distancing to services and professionals. Nonetheless, this result might be detailed across different types and sources of formal support other than those directed to schools and neighborhoods [4]. However, changes in daily routines seem to have been accompanied by changes in the support network, especially the importance of the informal network for positive discipline and the enriched environment, with parents feeling more supported, referring to the idea of social parenting advocated by Rodrigo [3]. These findings can undoubtedly be framed in the literature suggesting that parenting is molded through adjacent layers of personal and psychological characteristics of parents, child characteristics, interactions and relationships with intimate and extended family members and contextual circumstances. Thus, the inner world of parents, appraisals, emotions, interactions, namely interactions with the spouse or other family member, and institutional support are important factors to consider when discussing parenting [2,3].

Contrary to the previous findings regarding the impact of the employment condition on parenting dimensions, these results were not confirmed in the present study [13]. This might be due to differences from the original sample. In this study, we intended to limit the comparison to at-home parents (parents with governmental aids and teleworking parents). Although the change patterns between the two groups of parents were sufficiently informative of differences, these differences were not homogeneous and varied across all four domains of parenting, thus contrasting the previous findings [13].

Secondly, regression models enabled to test the differential effect of parental agreement and emotional regulation on the daily routine subscales. The findings indicated the importance of parental agreement and emotional regulation as predictors of positive discipline, parent–child activities, nurturing and an enriched environment. The observed causality of parents’ psychological characteristics and relational resources to parenting in daily life is a trace of the adaptative changes in different aspects of daily routines, and its critical role in a balanced and affectionate family’s functioning as well as for the parents and children’s well-being. Yet, regardless, it is possible to disentangle the weight and direction of specific factors—for instance, that emotional sensitivity predicts changes in positive discipline, nurturing and enriched environment, that emotional tension and emotional regulation impact parent–child activities, and that stress management predicts nurturing—these different predictors gain significance and expression in the whole context of good parenting. And the same can be said for other proximal or distal influencing factors. As shown, informal network was a predictor of positive discipline and enriched environment, while endorsement of new parent’s employment policy in COVID-19 times had an impact on parent–child activities and enriched environment. In the parenting process, these enhancing factors are clearly underlined by Rodrigo [3]. Highlighting the importance of team parenting and social support to parents as crucial levers to ease, enact and develop the parental roles across the entire life-span. It is important to mention the predictor role of the parent’s employment condition too, which revealed that at-home parents with governmental aids to care for their children felt more positive changes in their behavior, namely in parent–child activities and enriched environment than teleworking parents. These results demonstrated a higher burden felt by teleworking parents compared to at-home parents with government licensed work leave and aids. Regarding the demanding task of teleworking and the home-work balance during the COVID-19 pandemic have been published enhancing the need to pay more attention to the topic [13,16]. Remarkably, the parents’ employment condition was not a predictor of the positive discipline and nurturing, which might evidence the effort of parents to protect the relationship with the child. In fact, some studies revealed the importance of emotional regulation as a protective factor for children’s daily life during pandemic and to cope with the stress [5,6,7,8]. Even, in other studies, Portuguese parents, revealed that it, on the one hand, was hard to find the work–life balance, on the other hand, was a great opportunity to nurture and to bond family members [13,16]. All together, it might be said that beyond the specificity of each factor, their combined effect is reflecting the interconnection and the complexity of their influence as predictors in our model. This conclusion was addressed in our mediation model, finding an additional contribution to the explanation of direct influence of emotional experience and indirect influential path of support networks and co-parenting in family’s daily routines. The mediation model showed the important role that emotional experience can play in family dynamics. Other studies have already found results in the same line, whereas emotional regulation might be a protective factor to parenting, namely, parental stress, parents’ well-being, or parental burnout [5,7]. But co-parenting and family support are equally important to family dynamics, and they can be protective factors to parenting, parents’ well-being and to contribute to family resilience [10,11,12]. In fact, even if parents felt exacerbated by familial demands and emotional tension, these might not imply linear consequences on children’s adjustment, as a study with Portuguese and British parents illustrated [8]. Bearing upon the differential yet complex relation of mediating factors and processes involved in parent–child interaction and the family dynamics, also supports the family systemic perspective [2]. Additionally, the mediation model states a plausible explanation about different interfaces of daily routines and the direct and indirect influences of emotional experience and support network and co-parenting [2,3].

These results might outline an informative agenda when designing and programming family interventions, considering the nature and essence of parenting. Not solely the array of domains but the complexity and the dynamics of adaptative processes provide insightful recommendations for psychoeducational interventions and promotion of positive parenting and family resilience. Also, departing from lived experiences and concrete family situations in different countries and social realities might be suggestive of more specific and responsive interventions according to parents’ needs. Interventions that help the development of positive parenting and family resilience are crucial [23] as well as the continuity of research, adopting customary practices of a translational and evidence-based framework [24]. Worldwide, several studies documenting the research conducted during the pandemic have also tapped the importance of family support measures and of family-friendly policies, e.g., [25]. Most relevant is the support for family activities and practices, services, and policies that help families to overcome their vulnerabilities such as financial support or paid parental leaves [26], and multidisciplinary support that might be an asset to help family members if included the role of hope in the support process to promote the development of mental health [27].

Regarding post-pandemic interventions, these remain important as a societal and intergovernmental approach to face the consequences of the pandemic, strengthening the intersectorial delivery and developing their progressive universal delivery without compromising the implementation of specific measures for at-risk populations [28]. Looking at public health policymaking through the lens of social justice would promote social and public health policies that might overcome and prevent the occurrence of some difficulties and iniquities that were lived by some parents during the pandemic [22]. Further studies are still necessary to better comprehend the diverse consequences of the pandemic period on parenting as well as the effective interventions [26,29]. Doing so, a contribution to face similar future crises that may occur will be made. So, due to the importance of the pandemic consequences at the society level and families, and viewing families as a crucial social structure to future citizenship and societal development, “the family should be considered as an asset, an investment in the future, a social resource that is to be protected and supported, given its key role in the development of individuals and the protection of their rights, especially the most vulnerable, as well as an instrument for the social cohesion and welfare of communities” [3] (p. 67). In fact, interventions that can facilitate and stimulate adaptative parenting and family resilience are a need because they might strength families to cope with future crises and promote families’ well-being [20]. The development of systemic and multidisciplinary research models is fundamental to comprehend human resilience so its results might influence policies and practices and, consequently, “guide efforts by families, communities, societies, humanitarian agencies, and other human systems to prepare for, prevent, and/or respond effectively to circumstances and events that threaten human health and existence” [24].

Despite the contributions of this study, some limitations must be acknowledged. The instrument used to collect data was a self-report questionnaire, depending on personal perceptions. Data were collected online and the conditions about the questionnaire filling could not be controlled. Furthermore, participants did not represent the Portuguese population and generalization of the results is limited. Most participants were mothers, and their expectancies and attitudes facing motherhood, which were not measured, could influence the personal perceptions of changes and, consequently, the mediation model [30]. Also, at-risk families were not frequent or other specific groups such as single mothers and parenting dimensions such as coparenting can differ according to diverse family structures [31]. The social desirability of the participants’ responses was not controlled, thus future research should measure and control for social desirability response bias, adjusted for the specific types of social measures involved in the models [32].

Nonetheless, and to conclude, this study made contributions to the knowledge in the recent and novel condition related to COVID-19 lockdowns and families. We think the results might enlighten present and future studies, and interventions, dedicated to remedy, and to prevent, family crisis arising from stressful events such as the COVID-19 pandemic.

## Figures and Tables

**Table 1:** Descriptive statistics for the scales and subscales.

Scales and Subscales	N	M	SD	Range
Daily routine	878	3.30	0.469	1.77–5
Positive discipline	878	3.09	0.539	1.5–5
Parent–child activities	874	3.51	0.847	1–5
Nurturing	862	3.51	0.561	2–5
Enriched environment	866	3.13	0.739	1–5
Co-parenting	790	3.10	0.477	1–5
Parental alliance	783	3.02	0.490	1–5
Parental agreement	787	3.15	0.530	1–5
Emotional experience	802	3.56	0.439	1.75–5
Emotional regulation	800	3.33	0.580	1–5
Emotional sensitivity	782	3.44	0.596	1–5
Emotional tension	796	3.55	0.720	1–5
Stress management	789	4.12	0.765	1–5
Support network	777	3.07	0.602	1–5
Formal network	755	3.10	0.750	1–5
Informal network	775	3.02	0.676	1–5

**Table 2:** The Pearson’s correlation between employment condition, scales, and subscales.

	1	2	3	4	5	6	7	8	9	10	11	12	13	14	15	16	17
1. EC	-																
2. DR	0.152 **	--															
3. PD	0.095 **	0.813 **	--														
4. PCA	0.152 **	0.716 **	0.325 **	--													
5. N	0.062	0.580 **	0.311 **	0.342 **	--												
6. EEn	0.112 **	0.707 **	0.481 **	0.381 **	0.245 **	--											
7. CP	0.127 **	0.521 **	0.488 **	0.342 **	0.215 **	0.344 **	--										
8. PAg	0.140 **	0.516 **	0.475 **	0.334 **	0.223 **	0.354 **	0.960 **	--									
9. PAl	0.099 **	0.415 **	0.412 **	0.275 **	0.151 **	0.248 **	0.839 **	0.648 **	--								
10. EEx	0.001	0.396 **	0.317 **	0.188 **	0.373 **	0.301 **	0.333 **	0.319 **	0.277 **	--							
11. ES	0.089 *	0.546 **	0.458 **	0.308 **	0.403 **	0.416 **	0.470 **	0.465 **	0.384 **	0.715 **	--						
12. ET	−0.072 *	−0.110 **	−0.104 **	−0.191 **	0.080 *	−0.056	−0.089 *	−0.082 *	−0.110 **	0.574 **	0.017	--					
13. ER	0.075 *	0.515 **	0.409 **	0.371 **	0.311 **	0.374 **	0.443 **	0.401 **	0.425 **	0.692 **	0.606 **	0.034	--				
14. SM	−0.089 *	0.092 **	0.060	−0.010	0.196 **	0.064	0.048	0.073 *	−0.010	0.666 **	0.183 **	0.514 **	0.191 **	--			
15. SN	0.093 *	0.300 **	0.278 **	0.181 **	0.170 **	0.221 **	0.243 **	0.211 **	0.253 **	0.265 **	0.367 **	0.008	0.306 **	0.019	--		
16. FN	0.073 *	0.187 **	0.173 **	0.104 **	0.133 **	0.113 **	0.161 **	0.141 **	0.169 **	0.230 **	0.263 **	0.087 *	0.213 **	0.047	0.850 **	--	
17. IN	0.088 *	0.328 **	0.306 **	0.199 **	0.158 **	0.268 **	0.257 **	0.222 **	0.264 **	0.231 **	0.337 **	−0.050	0.309 **	−0.004	0.779 **	0.341 **	--

1. EC = Employment condition; 2. DR = Daily routine; 3. PD = Positive discipline; 4. PCA = Parent–child activities; 5. N = Nurturing; 6. EEn = Enriched environment; 7. CP = Co-parenting; 8. PAg = Parental agreement; 9. PAl = Parental alliance; 10. EEx = Emotional experience; 11. ES = Emotional sensitivity; 12. ET = Emotional tension; 13. ER = Emotional regulation; 14. SM = Stress management; 15. SN = Support network; 16. FN = Formal network; 17. IN = Informal network. ** *p* < 0.01; * *p* < 0.05.

**Table 3:** Multiple linear regression model predicting the positive discipline.

Predictors	R^2^	ΔR^2^	Standardized Coefficients (Beta)	t	*p*	95% IC	
						Lower Bound	Upper Bound
	0.323	0.314					
Employment condition			0.002	0.055	0.956	0.969	1.032
Parental agreement			0.281	6.364	<0.001	0.516	1.936
Parental alliance			0.050	1.144	0.249	0.520	1.922
Emotional sensitivity			0.188	4.401	<0.001	0.552	1.811
Emotional tension			−0.049	−1.302	0.193	0.715	1.398
Emotional regulation			0.119	2.864	0.004	0.582	1.719
Stress management			0.007	0.179	0.858	0.698	1.433
Formal network			0.008	0.224	0.823	0.851	1.175
Informal network			0.116	3.274	0.001	0.800	1.249

**Table 4:** Multiple linear regression model predicting the parent–child activities.

Predictors	R^2^	ΔR^2^	Standardized Coefficients (Beta)	t	*p*	95% IC	
						Lower Bound	Upper Bound
	0.230	0.220					
Employment condition			0.107	3.114	0.002	0.969	1.032
Parental agreement			0.169	3.586	<0.001	0.516	1.936
Parental alliance			−0.004	−0.082	0.935	0.520	1.922
Emotional sensitivity			0.011	0.245	0.807	0.552	1.811
Emotional tension			−0.169	−4.208	<0.001	0.715	1.398
Emotional regulation			0.311	7.005	<0.001	0.582	1.719
Stress management			0.025	0.614	0.539	0.698	1.433
Formal network			0.016	0.449	0.654	0.851	1.175
Informal network			0.040	1.056	0.291	0.800	1.249

**Table 5:** Multiple linear regression model predicting the nurturing.

Predictors	R^2^	ΔR^2^	Standardized Coefficients (Beta)	t	*p*	95% IC	
						Lower Bound	Upper Bound
	0.187	0.176					
Employment condition			0.040	1.118	0.264	0.970	1.031
Parental agreement			0.140	2.898	0.004	0.517	1.933
Parental alliance			−0.095	−1.967	0.050	0.521	1.919
Emotional sensitivity			0.233	4.966	<0.001	0.552	1.812
Emotional tension			0.028	0.681	0.496	0.715	1.398
Emotional regulation			0.109	2.393	0.017	0.582	1.718
Stress management			0.124	2.973	0.003	0.698	1.433
Formal network			0.027	0.717	0.474	0.851	1.175
Informal network			0.041	1.059	0.290	0.801	1.249

**Table 6:** Multiple linear regression model predicting the enriched environment.

Predictors	R^2^	ΔR^2^	Standardized Coefficients (Beta)	t	*p*	95% IC	
						Lower Bound	Upper Bound
	0.233	0.222					
Employment condition			0.101	2.942	0.003	0.970	1.031
Parental agreement			0.218	4.647	<0.001	0.520	1.923
Parental alliance			−0.055	−1.182	0.238	0.523	1.912
Emotional sensitivity			0.170	3.722	<0.001	0.550	1.817
Emotional tension			0.001	0.019	0.985	0.719	1.390
Emotional regulation			0.152	3.433	<0.001	0.586	1.705
Stress management			0.030	0.744	0.457	0.706	1.416
Formal network			−0.045	−1.234	0.218	0.847	1.181
Informal network			0.141	3.718	<0.001	0.801	1.248

**Table 7:** Mediation model to explain changes at daily routines.

					95% IC	
	Estimate	Standard Error	Z-Value	*p*	Lower Bound	Upper Bound
**Direct effects**						
Emotional experience → Daily routine	0.24	0.034	7.020	<0.001	0.154	0.317
**Indirect effects**						
Emotional experience → Support network → Daily routine	0.04	0.01	3.758	<0.001	0.019	0.066
Emotional experience → Co-parenting → Daily routine	0.15	0.020	7.852	<0.001	0.097	0.218
**Total effects**						
Emotional experience → Daily routine	0.43	0.035	12.231	<0.001	0.344	0.505
**Total indirect effects**						
Emotional experience → Daily routine	0.19	0.022	8.838	<0.001	0.130	0.258

## Data Availability

The data presented in this study are available upon request from the corresponding author.
